# Managing Cracked Teeth with Root Extension: A Prospective Preliminary Study Using Biodentine™ Material

**DOI:** 10.1155/2024/2234648

**Published:** 2024-05-09

**Authors:** Kênia Maria Soares de Toubes, Isabella Sousa Corrêa, Regina Célia Lopes Valadares, Stephanie Quadros Tonelli, Fábio Fernandes Borém Bruzinga, Frank Ferreira Silveira

**Affiliations:** ^1^Department of Dentistry, Uniube University, Uberaba, MG, Brazil; ^2^Department of Dentistry, Pontifical Catholic University of Minas Gerais, Belo Horizonte, MG, Brazil; ^3^Department of Dentistry, Faculty Arnaldo, Belo Horizonte, MG, Brazil; ^4^Department of Dentistry, José do Rosario Vellano University, Divinópolis, MG, Brazil

## Abstract

**Purpose:**

The authors of this study proposed an innovative approach involving the use of Biodentine™ material as an intraorifice barrier in cracked teeth with root extension to promote internal crack sealing, preventing the possibility of microinfiltration and apical crack propagation.

**Materials and Methods:**

The dental records of 11 patients with 12 posterior cracked teeth with root extension were included with a precise protocol performed by a senior endodontist. The treatment protocol included pulp diagnosis, crack identification using a dental operating microscope (DOM), endodontic treatment, placing a Biodentine™ as an intraorifice barrier, and immediate full-coverage restoration. The effectiveness of the treatment was assessed at two intervals, 6 months, and 1−3 years posttreatment, evaluating clinical, radiographic, and tomographic aspects. The treatment was deemed successful if there were no indications of radiolucency, sinus tracts, edema, or periodontal pockets associated with the crack line.

**Results:**

The study observed remarkably positive outcomes during the follow-up period, which spanned from 1 to 3 years. All the cracked teeth (100%) remained asymptomatic, meaning they were free of pain or discomfort. Furthermore, these teeth were in occlusal function. Both radiographic and tomographic assessments revealed the absence of bone loss along the crack line. This outcome signifies that the treatment effectively prevented further deterioration of the surrounding bone.

**Conclusions:**

Integrating advanced biomaterials and conservative restorative techniques has paved the way for innovative approaches in dental care. This protocol suggests a proactive step for managing cracked teeth with root extension. It addresses both biological aspects by sealing internal cracks and mechanical aspects by preventing crack progression, thereby improving these teeth' prognosis and long-term survival.

## 1. Introduction

The tooth is an exceptionally resilient structure, resistant to crack propagation under normal circumstances [1]. Even after sustaining a crack, a tooth can remain unaffected and symptom-free for a considerable period, often spanning several years [2, 3]. However, the behavior of a cracked tooth (CT) changes significantly when certain factors come into play, such as incorrect restoration, exposure to occlusal stress, parafunctional habits, or as a natural consequence of aging [4–6].

CTs typically exhibit one or more crack lines (CLs) that traverse through the tooth's structure and may establish connections with both the pulp and the periodontal ligament [4–6]. These CLs commonly extend in the mesiodistal direction and may involve one or both marginal ridges [4, 5]. However, the depth and severity of these CLs are clinically unpredictable and can lead to various pulp conditions, including reversible pulpitis, irreversible pulpitis, and pulpal necrosis [6, 7]. The decision to proceed with endodontic treatment hinges on the tooth's restorative potential [8, 9]. If a CT is not treated appropriately, the CLs may progressively deteriorate into a split tooth or develop severe periodontal defects [10]. Therefore, recognizing and effectively managing CTs is crucial for preserving their functionality and preventing more severe complications.

Although there is no consensus in the literature about the most effective treatment for a CT [11], early placement of a protective and adhesive restoration is highly recommended and is associated with a good prognosis for long-term treatment [6, 8]. This approach aims to seal and stabilize the tooth's structure, preventing further crack propagation and complications [8]. In contrast, CT's treatment with root extension and coexisting periodontal pocket are associated with an “unfavorable prognosis,” creating a dilemma regarding tooth survival [10, 12, 13].

Some authors claim that restorations do not fully protect CLs and are gradually recontaminated over time, leading to the spread of cracks, resulting in complete fractures and periodontal complications [10, 13]. However, new protocols for CTs with root extension have been suggested, with a promising longevity [14–17].

In this sense, a bioceramic material known as Biodentine™ (Septodont, Saint Maur des Fosse´s, France) [18] was used in this preliminary study as an intraorifice barrier in CTs with root extensions. Biodentine™ has demonstrated its versatility and effectiveness in various clinical settings, such as pulp capping, pulpotomy, irreversible pulpitis with full pulpotomy, retrograde filling, managing perforations, addressing resorption issues, and treating immature teeth with open apexes [19, 20]. Moreover, it has found application as a restorative material and a substitute for bioactive dentine [21, 22]. One of the remarkable characteristics of Biodentine™ is its ability to release calcium hydroxide upon contact with tissue fluids. This calcium hydroxide interacts with tissue phosphates, facilitating hydroxyapatite formation and inducing tissue regeneration [23, 24]. Notably, the crystallization of this material within the dentinal tubules creates a robust interface with the dentin. It contributes to enhanced resistance against microinfiltration, improved mechanical strength, and increased resistance to adhesion [21].

The literature review indicates that, up to this point, no study has explored the application of Biodentine™ as an intraorifice barrier in CTs with CLs extending to the root. Therefore, this preliminary prospective study pioneers the use of Biodentine™, preventing recontamination and halting the spread of cracks over time. The proposed approach revolves around three key steps: precise identification of crack extension, placement of Biodentine™ as an intraorifice barrier, and the immediate placement of an adhesive full-coverage restoration. By adopting this innovative protocol, the study aims to improve CT management and treatment outcomes with root-extending cracks.

## 2. Materials and Methods

The present prospective preliminary study followed the preexisting methodology for CTs [6, 25, 26] and was approved by the Research Ethics Committee of the Pontifical Catholic University of Minas Gerais, MG, Brazil (approval number: 4.427.989/2020). The study's data were collected from the clinical records of 11 patients treated from January 2020 to January 2023 at a private endodontic clinic.

### 2.1. Inclusion Criteria

The inclusion criteria were (1) the presence of posterior CT, (2) internal observation of radicular extension of the CL under a microscope, and (3) the extension of the CL should be observed at the level of the canal orifice and up to 3 mm beyond.

### 2.2. Exclusion Criteria

The exclusion criteria were (1) patients with CTs exhibiting a periodontal depth >6 mm associated with the CL, (2) CTs where the CL was limited to the coronary dentin or extended through the chamber floor, and (3) cases involving split teeth.

### 2.3. Nonsurgical Treatment Protocols

As part of the ethical considerations, all patients were thoroughly informed about the potential risks associated with the treatment, such as possible apical extension of the crack in short- and long-term and tooth loss, as well were presented with alternative treatment options such as immediate extraction and future placement of an implant.

Written informed consent was obtained from each patient to ensure their understanding and agreement with the proposed procedures. Radiographs and cone-beam computed tomographic (CBCT) images were acquired for each patient to provide comprehensive diagnostic information. Pulpal and periapical diagnoses were established based on these imaging modalities. A clinical inspection was also conducted, with periodontal probing depths ranging from 3 to 4 mm assessed. Any excursive occlusal interference causing discomfort was addressed, and necessary adjustments were made to alleviate occlusal symptoms. A local anesthetic agent was administered, and a rubber dam was placed. Carious lesions, amalgam, and definitive or temporary restorations were removed. The CL's number, location, and direction were determined using a dental operating microscope (DOM) (DF Vasconcelos, São Paulo, Brazil). This meticulous approach underscores the importance of comprehensive assessment and precise crack identification to guide the subsequent steps in the treatment protocol, ultimately contributing to the successful management of CTs.

All patients in the present study underwent two treatment sessions. The access cavity and root canals were performed in the first session with the RACE® EVO sequences 4% (Dentaire, La Chaux-de-Fonds, Switzerland). The root canals were irrigated with a total volume of 25 mL of 2.5% NaOCl (Lenza Pharma, Minas Gerais, Brazil); removal of debris and the smear layer was performed using 3 mL of 17% EDTA (Lenza Pharma, Minas Gerais, Brazil), followed by a final discharge with 2 mL of distilled water. The canals were dried with cell pack paper points. Under the guidance of a DOM, the internal depth of the crack was measured from the level of the canal orifice. This measurement was performed using a Hu-Friedy® periodontal probe (Hu-Friedy® Mfg. Co., Chicago, USA) with transillumination placed externally to the clamp wing. After these procedures, the canals were filled with a calcium hydroxide paste (Ultradent, South Jordan, Utah, USA), and the access cavity was temporarily restored using a direct composite splint (Resin Filtek Z 350) (3M ESPE, São Paulo, Brazil).

In the second treatment session, if no signs or symptoms of inflammation were present, the root canal treatment was concluded using AHP plus sealer (Dentsply Konstanz, Germany) and gutta-percha cones (Ultimate Dental, Tennessee, USA). The obturation materials were removed 2 mm apically to the deepest point of the radicular CL. The walls of the root canals were cleaned using ultrasound tips and dried with paper points. Subsequently, one capsule of Biodentine™ was mixed with 4–5 liquid drops for 30 s in an amalgamator at a speed of 4,000–4,200 rpm, and the mixed material was placed on a sterilized glass plate. Biodentine™ was inserted into the deepest CL as an extended orifice barrier with mineral trioxide aggregate (MTA) carrier and condensed using an endodontic condenser. The cavity was cleaned with distilled water after 15 min and sealed with a direct resin [27, 28]. One week later, the tooth received its definitive restoration. This restoration was a bonded full-coverage restoration fabricated using a computer-aided design and computer-aided manufacturing (CAD–CAM) system, all accomplished in a single step. This comprehensive treatment approach aims to effectively seal and stabilize the tooth structure, preventing further crack propagation and ultimately promoting the long-term survival and function of the treated tooth.

### 2.4. Nonsurgical Posttreatment Protocols

To ensure the long-term success and monitoring of the treatment outcomes, extra guidance, and night plates were provided to all patients. The follow-up process involved a combination of clinical, radiographic, and tomographic assessments.

#### 2.4.1. Clinical Examinations

Clinical examinations were performed at 6 months and 1−3 years after the initial treatment. These examinations included assessments for signs and symptoms, response to bite tests, and probing depth measurements.

#### 2.4.2. Radiographic Examinations

Radiographic images were reviewed to evaluate the presence of any periradicular radiolucency, which could be indicative of treatment failure.

#### 2.4.3. Tomographic Follow-Up

Tomographic follow-up was performed annually, providing a comprehensive assessment of the treated teeth over time.

**Figure 1:**
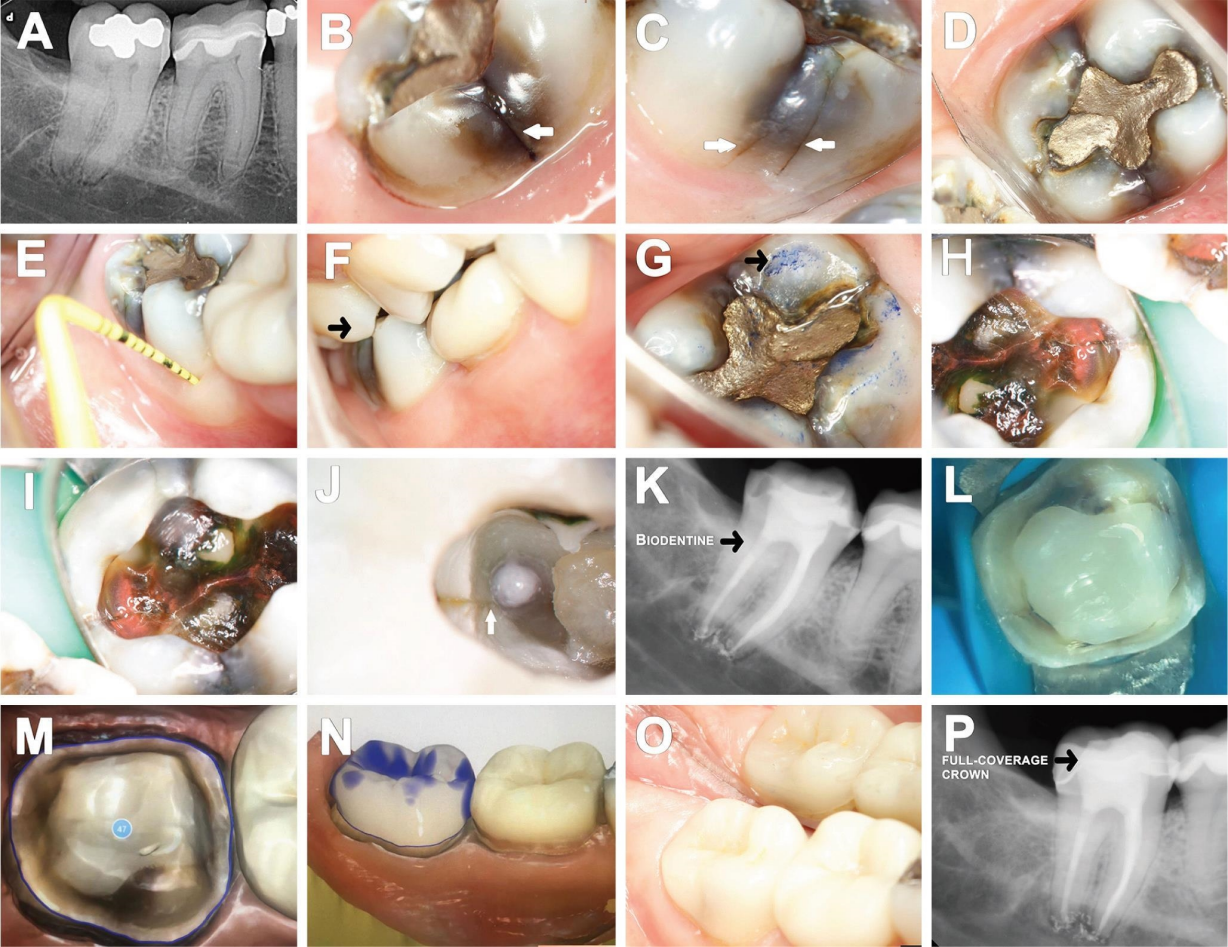
Initial digital radiography of tooth #31 shows symptomatic apical periodontitis (A); the presence of CLs in the lingual, buccal, mesial, and distal surfaces and class I amalgam restoration was identified under DOM (B–D); no periodontal probing depth was noticed (E); waste face and occlusal interferences were observed in the working movements (black arrows) (F, G); amalgam was removed, and CLs were assessed under DOM (H, I); internally, the crack line extended 2.0 mm beyond the level of the distal canal orifice (white arrow) (J); endodontic treatment was performed and Biodentine™ intraorifice barrier was placed 2.0 mm deepest the CL (black arrow) (K); preparation of full-crown coverage was performed and images from CEREC (Zurich, Switzerland) milling machine (L–N); bonded full-crown coverage in E-MAX (Ivoclar Vivadent, SP, Brazil) was fixed (O); final digital radiograph was taken after definitive restoration (P).

**Figure 2:**
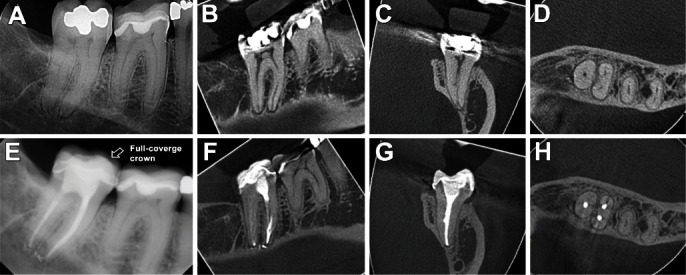
Initial digital radiography and CBCT images before treatment of tooth #31 (A–D); radiography and CBCT follow-up at 2 years, respectively (E–H).

**Figure 3:**
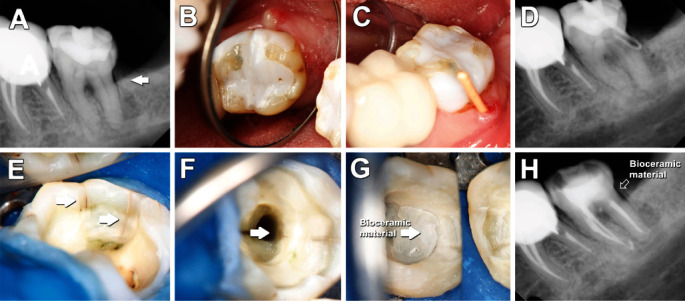
Initial digital radiography of tooth #18 shows asymptomatic apical periodontitis (A); the presence of buccal sinus tract localized in the one-third cervical of the tooth was noted (B); clinical and radiographic tracking of the sinus tract led to the cervical region via a distal approach (C, D); resin was removed, and CLs were assessed under the dental operating microscope in the distal marginal ridge extending 2.0 mm into the canal (white arrow) (E, F); endodontic treatment was performed, and Biodentine™ intraorifice barrier was placed 2.0 mm deepest the CL (white arrow); the coronary access was filled with resin for subsequent construction of a full-coverage crown bonded fixed (G, H).

**Figure 4:**
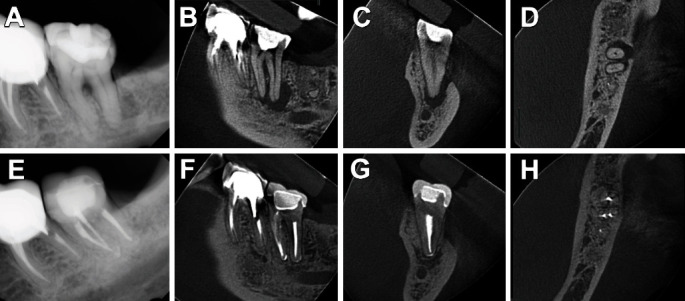
Comparative radiograph and CBCT images performed before (A–D) and after 2 years of treatment of tooth #18 revealed apical repair and the absence of crestal bone loss (E–H).

During these follow-up evaluations, specific indicators, such as pain, periradicular radiolucency, and increased probing depth, were carefully examined. The presence of any of these signs or symptoms could suggest treatment failure. This meticulous and multifaceted follow-up process allowed for the continuous monitoring of the treated teeth, enabling early detection of any potential issues and ensuring that appropriate measures could be taken promptly to maintain the long-term success and health of the teeth. Figures 1−4 illustrate the current treatment protocol used in this preliminary study.

## 3. Results

Categorical variables (periapical diagnosis, apical radioluscency, surface location of crack line, outcome) and numerical variables (crack depth orifice canal, probing depth, follow-up period) are described in Table 1.

The study involved a total of 12 CTs in 11 patients (nine women and two men). The patients' ages ranged from 52 to 74 years. Among these CTs, half were in the maxillary arch, comprising three molars and three premolars. Different pulpal and periapical diagnoses were made, with only one tooth previously treated. All the CTs in the study required endodontic intervention. Regarding the location of the CLs, approximately 33.4% of the CTs exhibited one CL in an isolated marginal ridge; 25.0% of the CTs had cracks on both the mesial and distal faces, the distal face was the most frequently affected, with 66.7% of the teeth having cracks on this face, either isolated or combined with other locations. The depth of the CLs extending apically ranged from 1 to 3 mm, with an average depth of 1.91 mm and a standard deviation of 0.90 mm. Probing depths ranged from 2 to 4 mm. Clinical and radiographic assessments were conducted during the follow-up period, which spanned from 1 to 3 years. These evaluations did not reveal any signs of treatment failure. Additionally, CBCT scans were performed pre- and post-treatment, indicating apical repair and an absence of crestal bone loss. These positive outcomes further emphasize the success and effectiveness of the treatment protocol used in managing the CTs in this study.

## 4. Discussion

The extent of the CL is an essential factor influencing the prognosis of endodontically treated CTs [29]. The margins of the restoration rarely encompass deep cracks; consequently, they become recontaminated or gradually propagate complete fractures [13, 29]. In the present prospective preliminary study performed on 12 CTs with root extension and followed up within 1−3 years, Biodentine™ was used as an intraorifice barrier to mitigate the risk of crack propagation by creating an environment that was not conducive to bacterial invasion.

Several strong points support this project's credibility and effectiveness in managing CTs with root extension. First, the use of modern technologies enables comprehensive assessment and treatment. Second, the treatments were carried out by the same experienced professional [6]. It is worth considering that, in initial studies with small samples, carrying out treatments by a single experienced professional can be an effective strategy to control external factors and obtain more reliable results, ensuring consistency and standardized care. Finally, the rapid restoration of CTs with full-coverage crowns using CAD–CAM technology contributes to long-term success and function [6, 26]. Additionally, in the present study, two high-resolution CBCTs were obtained for all patients pre- and post-treatment, allowing for accurate measurements of periapical radiolucency dimensions and angular defect extent [30]. The results showed that all patients (100%) remained symptom-free, and bone defects adjacent to CTs did not increase, as shown in Table 1.

Regarding the treatment plan for CTs with root extension, few studies could provide solid recommendations to be followed [13–16], and research is crucial to establishing effective treatment protocols. Some retrospective studies suggest an adhesive intracanal barrier as a solution, offering better sealing properties and greater resistance to fracture than traditional gutta-percha [31]. In one study of 87 CTs with root extension, CLs were sealed using flowable resin with a K file under a DOM, resulting in 68% survival and 53% success over 5 years [14]. Another study used bioactive flowable resin as an intraorifice barrier, achieving a high survival rate of 100% at 2 years and 96.6% at 4 years of follow-up [17].

Other available data come from observational studies, case reports, and in vitro investigations. Although these studies are valuable for generating hypotheses and insights, their nonrandomized and often anecdotal nature introduces a high potential for bias in their findings [15, 16]. Michaelson proposed a radical approach involving three CTs with root extension. This technique involved completely removing CLs using a drill or an ultrasonic tip, creating a coronal–radicular perforation sealed with MTA. The aim was to eliminate the source of the crack and seal the perforation with a biocompatible material [15]. Mahgoli et al. [16] conducted a study on five CTs previously treated with cracks in the pulp chamber floor. They used PANAVIA™ cement to seal the CLs and observed positive outcomes during a 10-year follow-up. Notably, none of the abovementioned studies [14–17] incorporated CBCT in their methodologies. The incorporation of CBCT in managing and monitoring CTs with root extension could be a good approach to prevent false-negative diagnoses [30].

The placement of intracanal posts in CTs with root extension should be carefully evaluated on a case-by-case basis, as they can contribute to tooth loss and periodontal destruction [6, 10]. Then, intrabarriers made of bioceramic or bonded materials can be a safer alternative for CTs with root extension [14–17]. Studies reported that flowable adhesive materials, Biodentine™, and MTA demonstrate better sealing capacity than gutta-percha. This superior sealing ability is crucial in preventing bacterial infiltration and crack propagation [14, 16]. In this sense, Biodentine™ offers several advantages over MTA, including good chemical adhesion to dentin, high compressive strength, minimal tooth discoloration, and shorter setting time [32, 33]. Thus, it is more bioactive and biocompatible than adhesive intraorifice barriers [20, 34, 35]. Biodentine™ is often called an “all-in-one first” material [22], indicating its versatility in different dental applications. It can be advantageous in deep cracks where photoactivation and adhesion may be challenging due to its chemical adhesion properties.

In the current study, Biodentine™ was chosen based on its elasticity, aiming for a similar modulus as dentin to enhance stress distribution at the crack site. These approaches offer innovative solutions to address the challenges of CTs with root extension. However, further research and clinical validation are required before widespread adoption. It is important to consider each CT case individually, taking into account the patient's condition and risk factors when making treatment decisions.

One of the main limitations of this study was the relatively small sample size, which restricts our ability to perform a more comprehensive and statistically robust analysis. However, preliminary studies like this are vital in proposing new treatment approaches and generating hypotheses for future research. In addition, it is worth mentioning that exploring new experimental models that can evaluate the impact of the intraorifice barrier on crack propagation may represent a significant path for further investigation. Considering our limitations, we recommend future studies with a larger sample and involvement of multiple providers or centers may provide a more comprehensive and representative view of the effectiveness of the protocol. The diversity of professionals and clinical settings can help assess the generalizability of results and identify possible variations in treatment application.

## 5. Conclusions

The biological and mechanical management of CTs with root extension led to the formulation of a reliable and replicable therapeutic approach based on three fundamental principles:

- Identification of crack extension: This initial step involves precisely identifying the apical crack extension under DOM.
- Placement of barrier with bioceramic material: This barrier should extend at least 2 mm beyond the crack. This bioceramic material helps seal and stabilize the crack internally, preventing long-term recontamination.
- Immediate placement of full-coverage crown: As the final step in this therapeutic strategy, this crown is a protective measure to stabilize and paralyze the propagation of the apical crack over time.

In summary, combining Biodentine™ as an intraorifice barrier, precise crack identification, and conservative restorative techniques represents a promising strategy for managing CTs. This approach addresses the immediate concerns and promotes the long-term survival and success of treatment for these challenging cases.

## Figures and Tables

**Table 1:** The characteristics of the patients treated by the proposed protocol and the follow-up time and outcome.

Categorical variables	*n* (%)
Periapical diagnosis	
Symptomatic irreversible pulpitis	8 (66.7%)
Asymptomatic irreversible pulpitis	1 (8.3%)
Pulp necrosis	2 (16.7%)
Previously treated	1 (8.3%)
Apical radioluscency	
Present	7 (58.3%)
Absent	5 (41.7%)
Surface location of crack line	
Distal	4 (33.4%)
Disto-bucal	1 (8.3%)
Mesial-distal	3 (25.0%)
Mesial-buccal	1 (8.3%)
Mesial	1 (8.3%)
Palatal	2 (16.7%)
Outcome	
Failure	0 (0.0%)
No failure	100 (100.0%)

Numerical variables	Value

Crack depth orifice canal (mm)	
Mean (standard deviation)	1.91 (0.90)
Min–max	1–3
Probing depth (mm)	
Mean (standard deviation)	2.83 (0.71)
Min–max	2–4
Follow-up period (years)	
Mean (standard deviation)	1.91 (0.90)
Min–max	1–3

## Data Availability

The data used to support this study are available from the corresponding author upon request.
